# A honeycomb-like structure in the left anterior descending coronary artery treated using a scoring device and drug-eluting stent implantation: a case report

**DOI:** 10.1186/s13256-016-0874-y

**Published:** 2016-04-01

**Authors:** Tatsuo Haraki, Ryota Uemura, Shin-ichiro Masuda, Nobuhiko Kobayashi, Takeshi Lee

**Affiliations:** Department of Cardiology, Saitama Eastern Cardiovascular Hospital, Osawa 3187-1, Koshigaya, 343-0025 Saitama Japan

**Keywords:** Honeycomb-like structure, Coronary artery, Scoring devices, Stent implantation, Apixaban

## Abstract

**Background:**

A honeycomb-like structure in the coronary artery is rarely diagnosed by intracoronary ultrasound or optical coherence tomography. Further, its structural mechanisms and response to interventional therapy remain unknown.

**Case presentation:**

A 59-year-old Japanese man was referred to our hospital because of acute decompensated heart failure with rapid atrial fibrillation. After receiving anticoagulant therapy, a coronary angiogram revealed a braid-like appearance and an intracoronary ultrasound image confirmed a honeycomb-like structure in the mid left anterior descending coronary artery. We inserted two guide wires into different partitions. Although a balloon angioplasty with a scoring device could not completely fenestrate these partitions, a stent implant was able to completely compress the structure easily.

**Conclusions:**

The honeycomb-like structure of the left anterior descending coronary artery in our patient was suspected to be because of recanalization of a cardiogenic embolism. This structure may have been composed of relatively hard tissues, but was easily compressed by a stent implantation.

## Background

There are several case reports of a honeycomb-like structure (or lotus root-like appearance) observed in the coronary artery of patients with Kawasaki disease, antiphospholipid syndrome, embolic stroke, atrial fibrillation, and acute coronary syndrome [1–7]. However, its structural mechanisms and response to interventional therapy remain unknown. We report the case of a patient with a honeycomb-like structure in the mid left anterior descending coronary artery (LAD), which was treated by intervention using a scoring device and implantation of a drug-eluting stent.

## Case presentation

**Fig. 1:**
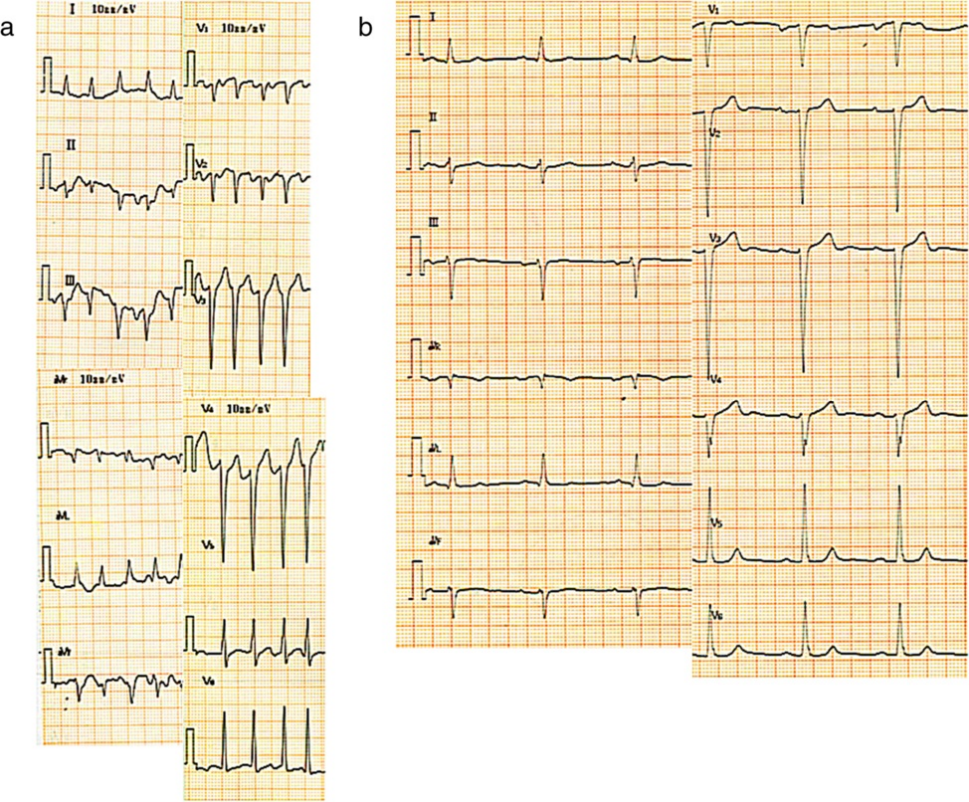
Electrocardiogram demonstrating rapid atrial fibrillation with poor R wave progression in leads V_1_–V_4_ (**a**). After 3 days, our patient obtained sinus rhythm, and an electrocardiogram showed left axis deviation and poor R wave progression in leads V_1_–V_4_ without ST-T change (**b**), which was suspected to represent a complication arising from a previous myocardial infarction

**Fig. 2:**
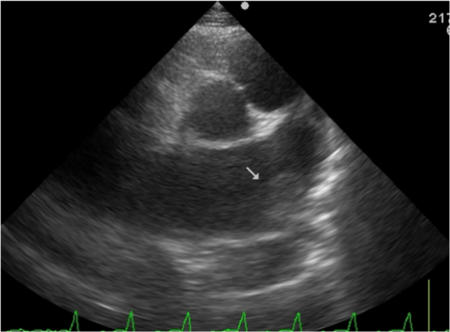
Echocardiograph demonstrating a thrombus-like echo in the left atrium (*white arrow*)

A 59-year-old Japanese man, a current smoker, had experienced shortness of breath for a few days and was referred to our hospital because of palpitations and paroxysmal nocturnal dyspnea. He had no clinical history of heart failure and had not been receiving any medications. Upon admission, he was tachycardic (approximately 150–180 heartbeats/minute) with stridor, desaturated with an atrial oxygen saturation (SaO_2_) of approximately 97 % (on 4 L oxygen/minute) via an oxygen mask, and normotensive with a blood pressure of 126/91 mm Hg. Furthermore, he exhibited mild peripheral edema. An electrocardiogram (ECG) demonstrated rapid atrial fibrillation and poor R wave progression in leads V_1_–V_4_ (Fig. 1a). A chest X-ray demonstrated cardiomegaly with a cardiothoracic ratio of 62 % along with pulmonary congestion and pleural effusion. His NT-proBNP level was significant at 7760 pg/mL and his troponin T level was slightly elevated at 0.047 ng/mL. His CK-MB level was normal at 10 U/L. Echocardiography demonstrated global left ventricular hypokinesis and akinesis in the anteroseptal to apical wall. The left ventricular end-diastolic dimension was 59 mm, whereas the left ventricular ejection fraction (LVEF) was decreased to 20 %. The left atrium was slightly dilated at 43 mm and a thrombus-like echo was evident (Fig. 2). According to the Framingham criteria, he was diagnosed with acute decompensated heart failure with rapid atrial fibrillation. We suspected further complication with acute coronary ischemia.

Initially, we intravenously administered diuretics, carperitide (0.033 μg/mL/minute), digitalis, and heparin (12000 U/d). By the following day, the atrial fibrillation had converted to an atrial flutter (2:1 to 4:1 conduction); however, the electrocardiogram showed no ST-T change except for poor R wave progression in leads V_1_–V_4_. In addition, troponin T (0.045 ng/mL) and CK-MB (6 U/L) levels were not elevated on the following day. We considered that there were no complications arising from acute coronary syndrome, and therefore, we continued the conservative treatment. Our patient required additional medication, including diltiazem and bisoprolol, to control the rate of the atrial flutter.

**Fig. 3:**
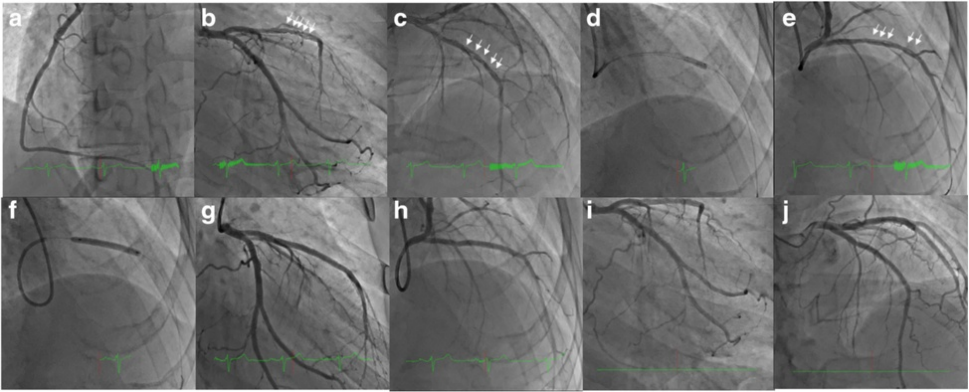
Coronary angiogram 11 days after admission. The right coronary artery appeared normal (**a**). The left circumflex coronary artery was also normal; however, the mid left anterior descending coronary artery showed a braid-like appearance (**b**, **c** *white arrows*). Balloon inflation was performed using a scoring device and a buddy wire (**d**). A braid-like appearance was observed even after inflating the balloon with a scoring device (**e** *white arrows*). An everolimus-eluting stent was subsequently implanted with nominal pressure (10 atm) (**f**). The honeycomb-like structure was completely compressed following stent implantation (**g**, **h**). After 14 months, we examined the stent implantation site in the mid-LAD and confirmed that there had not been a recurrence of stenosis (**i**, **j**)

**Fig. 4:**
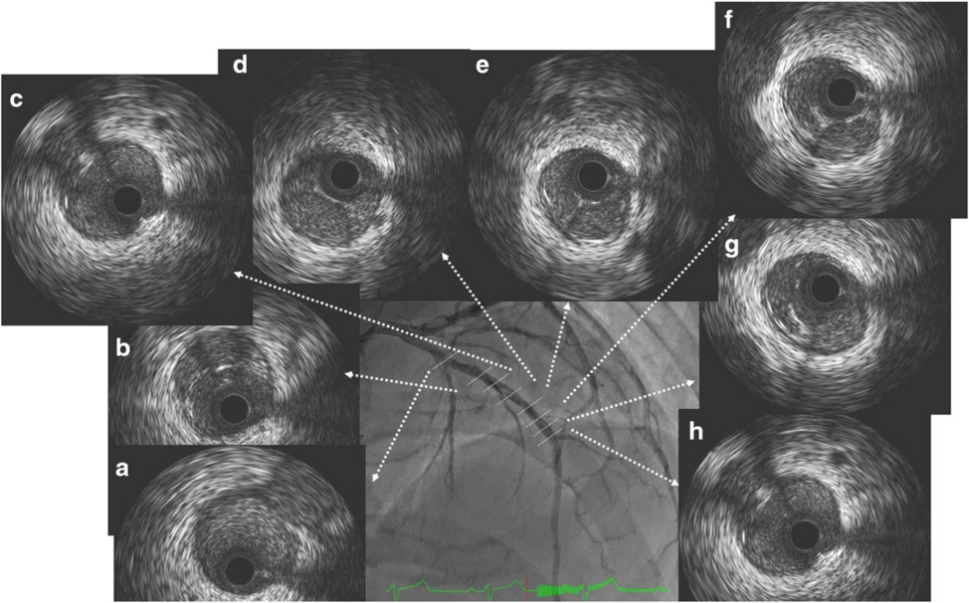
Intravascular ultrasound image demonstrating the honeycomb-like structure in the mid left anterior descending coronary artery (**b**–**h**). No atheromatous plaques were seen (**a**)

**Fig. 5:**
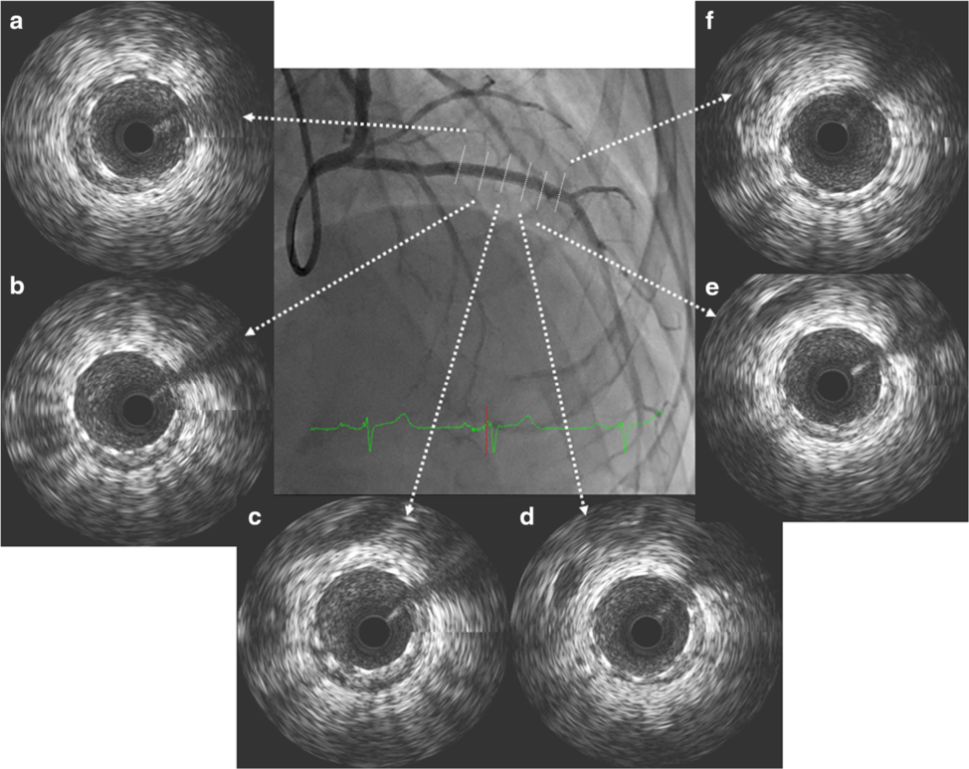
Intravascular ultrasound image demonstrating that the honeycomb-like structure in the mid left anterior descending coronary artery had been compressed by stent implantation (**a**–**f**)

After 3 days, the atrial flutter spontaneously converted to a sinus rhythm. The electrocardiogram revealed left axis deviation and poor R wave progression in leads V_1_–V_4_ without ST-T change (Fig. 1b). We suspected that this was a complication caused by prior myocardial infarction in the anteroseptal wall. After the sinus rhythm returned, his symptoms, including shortness of breath and palpitations, were clinically improved. We subsequently changed from a continuous intravenous injection of heparin to an oral dose of 10 mg of apixaban. A chest X-ray, which was performed 5 days after admission, showed no lung congestion or pleural edema, and the cardiothoracic ratio was also improved to 50 %. Thallium-201/iodine-123-15-(p-iodophenyl)-3-(R,S)-methylpentadecanoic acid dual single-photon emission computed tomography revealed ischemia in the left ventricular anterior to apical wall. Therefore, we performed a coronary angiogram (CAG) 11 days after admission. CAG demonstrated that the right coronary artery (Fig. 3a) and left circumflex coronary artery were normal; however, the left anterior descending coronary artery (LAD) showed a braid-like appearance (Figs. 3b, c). An intravascular ultrasound (IVUS) image (with a pullback speed of 0.5 mm/seconds) demonstrated a honeycomb-like structure midway along the LAD, which was divided by several high and low echoic partitions (Figs. 4b–h). There were no atherosclerotic plaques or intramural hematomas. Percutaneous coronary intervention of the mid-LAD was performed by inserting two guide wires into different partitions. Balloon angioplasty with a scoring device (Scoreflex™, OrbusNeich Medical, Tokyo, Japan) and buddy wire could not completely fenestrate these partitions (Fig. 3e). Therefore, we implanted a 3.5 × 33 mm everolimus-eluting stent (Xience Alpine®, Abbott Vascular, Tokyo, Japan) with nominal pressure (10 atm). This structure was easily completely compressed following stent implantation (Figs. 3g, h), which was additionally confirmed by IVUS (Fig. 5).

After 14 days of admission (prior to discharge), a repeat echocardiogram confirmed the persistence of global left ventricular hypokinesis with an LVEF of 19 %. We managed this condition by administering aspirin, 100 mg; clopidogrel, 75 mg; apixaban, 5 mg; furosemide, 40 mg; spironolactone, 25 mg; digoxin, 0.125 mg; metoprolol, 2.5 mg; and perindopril, 4 mg.

The sinus rhythm was maintained for 11 months following stent implantation, and there were no events relating to ischemic or decompensated heart failure during this time. After 11 months, another echocardiogram showed moderate hypokinesis in anteroseptal and inferior walls, a left ventricular end-diastolic dimension of 57 mm, and it confirmed that LVEF had improved to 44 %. After 14 months, a repeat CAG confirmed that there had been no recurrence of stenosis at the stent implantation site in the mid-LAD, and no other stenotic lesions were evident (Figs. 3i, j).

## Discussion

Several case reports have indicated that the honeycomb-like structure (or lotus root-like appearance) observed in the coronary artery by IVUS or optical coherence tomography (OCT) is caused by a spontaneous recanalization of thrombi (for example, an organized thrombus) [3–5]. An organized thrombus includes new capillary vessels and hyperplasia of the elastic and collagen fibers, which results in thrombi. OCT revealed that the septa in the honeycomb-like structure was composed of a high signal intensity and a low signal attenuation, suggesting that it consisted of a fibrous material that represented the recanalization of a cardiogenic embolism. In our patient, this structure was mixed with high and low echoic partitions by IVUS. It was suspected that the degree of fibrosis may be related to the echogenicity [2]. This structure could not be completely fenestrated by a balloon angioplasty with a scoring device, even with the buddy wire technique. However, stent implantation did completely compress this structure, indicating that it was likely composed of relatively hard tissues, which may have included fibrous material. Although we did not evaluate this structure histologically, from a clinical perspective, this honeycomb-like structure of the LAD is thought to be due to cardiogenic thrombus recanalization rather than plaque erosion. It is reported that the resolution of left atrial appendage thrombus was observed with apixaban, a member of the class of novel oral anticoagulants (NOAC) [8]. We performed IVUS after 9 days of apixaban treatment. Apixaban usage may have modified the tissue characteristics of this honeycomb-like structure.

Furthermore, it has been reported that a lesion with a lotus root-like appearance in stable coronary artery disease was functionally severe stenosis, identified by myocardial fractional flow reserve and coronary flow velocity reserve indexes [9]. In our patient, the sinus rhythm was maintained for 11 months after stent implantation, and a repeat CAG confirmed that there had been no recurrence of stenosis at the stent implantation site in the mid-LAD, however, the LVEF was not fully improved (44 % by echocardiograph). Further careful follow-up is needed to check his cardiac function as well as to avoid the recurrence of atrial fibrillation and/or embolic events.

## Conclusions

We present the case of patient with a honeycomb-like structure in the LAD that was suspected to be because of recanalization of a cardiogenic embolism. This structure may have been composed of relatively hard tissues, but was easily compressed by stent implantation.

## Consent

Written informed consent was obtained from the patient for publication of this case report and any accompanying images. A copy of the written consent is available for review by the Editor-in-Chief of this journal.
